# Factors related to mortality in critically ill histoplasmosis: a multicenter retrospective study in Guadeloupe and French Guyana

**DOI:** 10.1186/s13613-023-01128-7

**Published:** 2023-04-21

**Authors:** Laurent Camous, Arthur Surel, Hatem Kallel, Muriel Nicolas, Frederic Martino, Marc Valette, Alexandre Demoule, Jean-David Pommier

**Affiliations:** 1 Intensive Care Unit, Guadeloupe Teaching Hospital, Antilles-Guyane University, Chemin de Chauvel, Les Abymes, France; 2 Intensive Care Unit, Cayenne Hospital, French Guyana, France; 3 Mycology Department, Guadeloupe Teaching Hospital, Antilles-Guyane University, Chemin de Chauvel, Les Abymes, France; 4 Université de Paris and Université des Antilles, INSERM, BIGR, 75015 Paris, France; 5 UMRS1158 Neurophysiologie Respiratoire Expérimentale et Clinique, Sorbonne Université, INSERM, 75005 Paris, France; 6 Service de Médecine Intensive et Réanimation (Département R3S), AP-HP, Groupe Hospitalier Universitaire APHP-Sorbonne Université, Site Pitié-Salpêtrière, 75013 Paris, France; 7 Institut Pasteur de Guadeloupe, Morne Jolivière, 97139 Les Abymes, France; 8 Réanimation médicale et chirurgicale–CHU de Guadeloupe, 97139 Les Abyme, France

**Keywords:** Histoplasmosis, Intensive care unit, Fungal infection, Immunodeficiency, AIDS

## Abstract

**Purpose:**

To describe clinical and biological features and the outcomes of patients admitted for histoplasmosis in two intensive care units (ICU) in French Guyana and in the French West Indies (Guadeloupe).

**Methods:**

All patients admitted to these two ICUs for culture-proven histoplasmosis between January 2014 to August 2022 were included in the study. Using univariate and multivariate analysis, we assessed risk factors at ICU admission that were associated with death.

**Results:**

Forty patients were included (65% men). Median age was 56 years and simplified acute physiologic score (SAPS) II was 65. HIV was found in 58%, another immunodeficiency was identified in 28%, and no underlying immunodeficiency could be identified in 14% of patients. Within the first 24 h of ICU admission, 85% of patients had acute respiratory failure, 78% had shock, 30% had coma, and 48% had hemophagocytic lymphohistiocytosis. Mechanical ventilation was instituted in 78% of patients and renal replacement therapy in 55%. The 30-day mortality was 53%. By multivariate analysis, factors independently associated with 30-day mortality were SOFA score (odds ratio [OR] 1.5, 95% confidence interval [CI] [1.1–2.1]), time between symptom onset and treatment per day (OR 1.1, 95% CI 1.0–1.1), and hemophagocytic lymphohistiocytosis (OR 6.4, 95% CI 1.1–47.5).

**Conclusion:**

Histoplasmosis requiring ICU admission is a protean disease with multiple and severe organ involvement. Immunodeficiency is found in most patients. The prognosis remains severe despite appropriate treatment and is worsened by late treatment initiation.

**Supplementary Information:**

The online version contains supplementary material available at 10.1186/s13613-023-01128-7.

## Background

*Histoplasma capsulatum* *var. capsulatum* is a dimorphic fungus endemic in South America, the Caribbean area, and North America [1, 2] Histoplasmosis usually results from inhalation of aerosolized spores, with an estimated 1% of infections resulting in respiratory symptoms [2]. In the American continent and in subtropical areas worldwide, *Histoplasma spp.* are highly prevalent. In the French West Indies and French Guiana, up to 30% of the population is thought to have been exposed to *Histoplasma capsulatum* [3, 4]. Histoplasmosis with systemic involvement was first described in the early 1980s [5], even though the underlying risk factors were unknown during this period. Later, it was reported in immunocompromised hosts, including HIV-infected patients with acquired immune deficiency syndrome (AIDS) [1]. During the same decade in the United States [1], histoplasmosis was reported as one of the most common opportunistic infection in AIDS patients [6]. According to EORTC and ATS classifications [7–10], histoplasmosis can be pulmonary [9] (only affecting the lungs) or disseminated [11] with positive blood cultures and invasion of another organ [2, 5]. In large epidemiological cohorts, histoplasmosis is disseminated in up to 90% of the immunocompromised hosts [1, 2, 5, 12] and is reported to be more severe [1]. In AIDS patients, outcome seems grim, with frequent intensive care unit (ICU) admission [13] and a high mortality rate (from 40% up to 71%) [14]. For other immunocompromised hosts, epidemiological data remain scarce [7, 15]. The characteristics and prognosis of critically ill patients with histoplasmosis requiring an ICU admission are not well reported.

The purpose of the present study was to describe clinical, biological features and outcome at Day 30 of patients admitted to the ICU for histoplasmosis in French Guyana and in French West Indies (Guadeloupe) over an 8-year period. In addition, we compared the characteristics of patients according to the underlying cause of immunosuppression. Finally, we aimed to identify factors associated with 30-day mortality.

## Methods

### Study design

This retrospective bicentric study was conducted in two ICUs, the Guadeloupe University Hospital (GUH, Guadeloupe Island, French West Indies; 30 beds) and the Cayenne Hospital (French Guyana; 18 beds) from January 2014 to August 2022. The study was approved by the local ethics committee, which waived consent for retrospective anonymous data collection according to French Law, and it was registered under the number 2223746 v 0. This study follows the Strengthening the Reporting of Observational Studies in Epidemiology (STROBE) statement.

### Patient selection

Cases were identified through computerized medical records and laboratory microbiology databases of each institution. All consecutive adult patients (> 18 years old) admitted to the ICU for proven histoplasmosis were screened for inclusion.

Criteria for histoplasmosis were [1] symptoms consistent with fungal infection such as fever, respiratory or gastrointestinal or central nervous system manifestations, skin lesions, lymphadenopathy and hepatosplenomegaly; with [2] either a positive culture of body fluid samples (bronchoalveolar lavage, blood culture, or bone marrow culture) with histopathologic demonstration of morphologic forms consistent with *Histoplasma capsulatum* by Gomori methenamine silver stain culture [7] or a positive RT-PCR. Classification of fungal infection was done following EORTC (European Organization for Research and Treatment of Cancer) and ATS guidelines [8–10].

### Data collection and syndrome definitions

Age, SOFA score, geographic origin, and sex were collected at ICU admission. The underlying condition favoring histoplasmosis was classified as follows: [1] HIV-infected patients [2]. Patients with other immunodeficiency included solid organ transplant (SOT) recipients; patients receiving immunosuppressive therapy such as steroids (> 20 mg/day), anti-TNF, or azathioprine, and those with hematological malignancy; and [3] patients with no identified immunodeficiency.

Clinical characteristics within the first 24 h of ICU admission were collected as follows: body temperature, neurological symptoms (confusion: an altered state of consciousness or a focal neurological deficit with no alternative diagnosis), gastrointestinal symptoms (diarrhea, abdominal pain, or colitis on abdominal computed tomography with no alternative etiology) and respiratory symptoms (cough, hemoptysis, or shortness of breath).

Microscopic examination from broncho-alveolar lavage (BAL), Bone marrow aspirate, and blood were collected. Bacterial and viral concomitant infections diagnosed at ICU admission with biological fluid analysis and cultures (blood samples, bronchoalveolar lavage, urine samples) were also collected.

All radiological data (cerebral, thoracic, and abdominal CT) were collected and reviewed. When thoracic CT was performed, we reported the presence of macro-nodules, adenopathy, and miliary pattern (defined as multiple small (< 4 mm) pulmonary nodules [16]).

Severe organ involvement within the first 24 h of ICU admission was defined as follows:

Coma was defined as a Glasgow Coma Scale score < 8.

Acute respiratory failure (ARF) was defined by an oxygen flow ≥ 5 L/min to maintain SpO_2_ > 92% with respiratory rate > 30/min.

Shock was defined by mean arterial pressure below 65 mm Hg associated with a lactatemia of > 2 mmol/L and need for vasopressors [17].

Hemophagocytic lymphohistiocytosis (HLH) was defined according to the Saint Antoine criteria [18].

Histoplasmosis was classified according to the EORTC and ATS criteria [8–10]:

-Histoplasmosis was pulmonary when *Histoplasma spp.* was isolated only from bronchoalveolar lavage with no other sign of extrapulmonary infection.
-Histoplasmosis was disseminated when *Histoplasma spp*. was isolated from at least another site than lung, with or without lung involvement.

Advanced life support therapy taken during the ICU stay (such as invasive mechanical ventilation, vasopressor support, and renal replacement therapy [19]), the nature of antifungal therapy used for histoplasmosis treatment [7], and the chronology of main events (time before diagnosis and ICU admission, time between ICU admission and diagnosis) were determined for each patient. Finally, 30-day mortality and length of ICU stay were collected.

### Statistical analysis

Analyses were performed using R (version 4.0.4) [20]. Continuous variables were reported as median (interquartile range) and categorical data as number (percent). HIV-infected patients, those with other immunodeficiency, and patients without any known underlying immunodeficiency were compared using Fisher’s exact tests for categorical variables and Kruskal–Wallis for continuous variables. Survivors and nonsurvivors 30 days after ICU admission were compared with the Fisher’s exact tests for categorical variables and the Mann Whitney test for continuous variables. Logistic regression analysis was used to identify the variables significantly associated with 30-day mortality. Variables yielding P values < 0.10 in the univariate analyses were entered in a stepwise procedure. In case an assessment score was entered in the multivariate model (e.g., SOFA), variables assessed by the score were not entered in the model. We then did backward selection on this model, stopping when the Akaike information criterion reached its minimum. A sensitivity analysis to assess variables associated with 30-day mortality was also performed only in immunocompromised patients (including HIV-infected and other immunodeficiency patients) after excluding patients without any identified immunodeficiency. Kaplan–Meier overall survival curves up to day 30 were separately computed for relevant variables and were compared using log-rank tests.

## Results

### Patients’ selection and criteria for Histoplasmosis

**Fig. 1:**
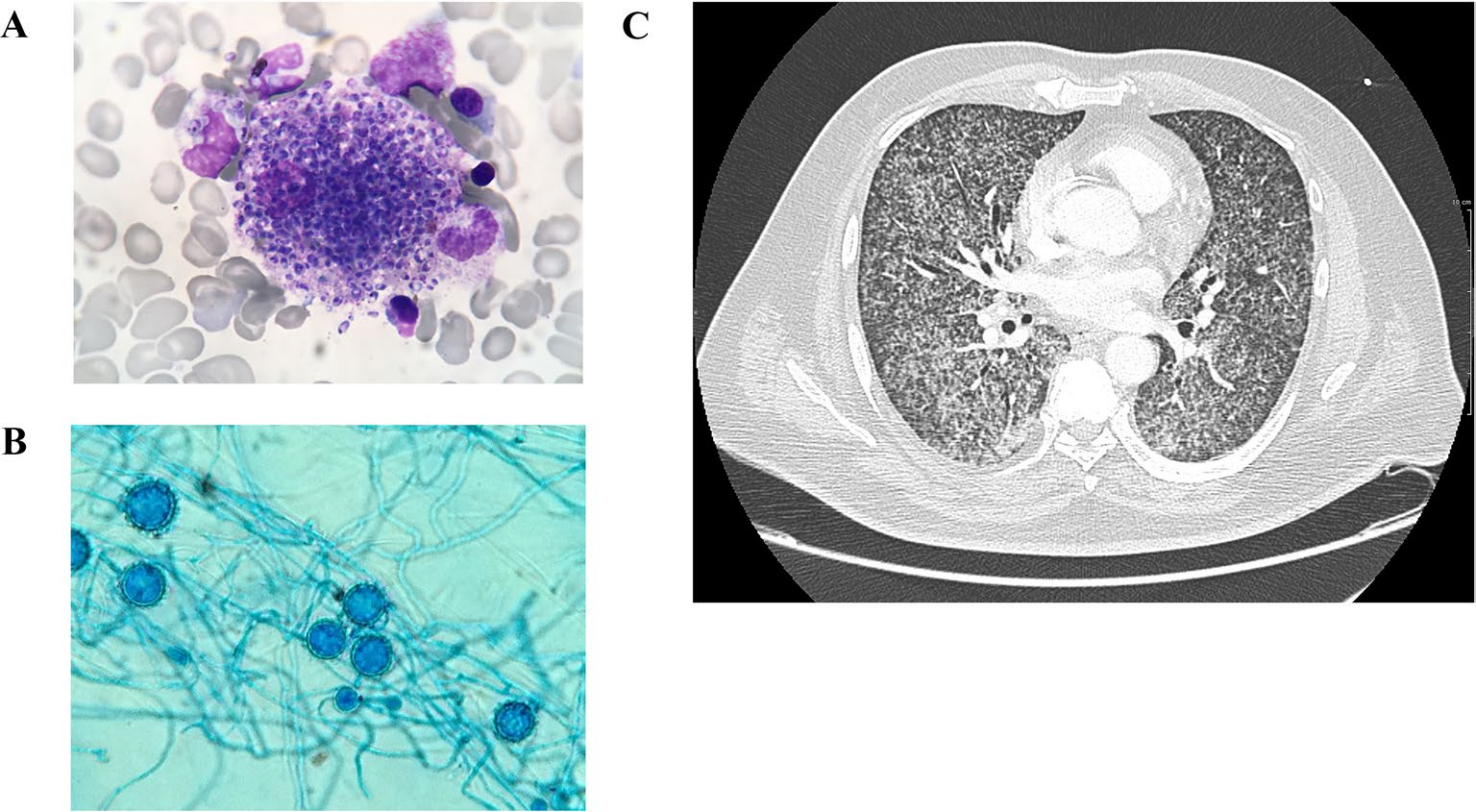
Microbiological analysis and tomodensitometry in a patient with disseminated histoplasmosis. **A** Shows Gomori–Grocott staining of intracellular *Histoplasma capsulatum* in macrophage (medullogram). **B** Shows Gomori–Grocott staining in a culture of bronchoalveolar lavage. **C** Shows a thoracic CT scan with a diffuse miliary pattern.

During the study period (2014–2022), 86 patients were admitted for histoplasmosis in the two centers. Among them, 40 were admitted in the two participating ICUs: 13 in French Guyana and 27 in Guadeloupe. Histoplasmosis was microbiologically proven in all patients. Table 1 describes the samples on which the microbiological diagnoses were performed. Yields of biological fluid analysis (direct examination and cultures) are reported in Table 1. The best yield for *Histoplasma capsulatum* identification was from bronchoalveolar lavage (Fig. 1A). Bone marrow aspirate analysis, when performed, also had a high yield of *Histoplasma capsulatum* identification (Fig. 1B). The diagnosis was performed before ICU admission in 6 patients (15%) and during the ICU course for the remaining 34 patients (85%). Four (10%) patients died before the culture was positive. Of note, RT-PCR was performed on the bronchoalveolar lavage of 7 patients and was positive in all of them (3 out of 7 had a negative direct examination and all had a positive culture).

**Table 1:** Mycological diagnosis

	All *n* = 40
Positive bronchoalveolar lavage samples	
Direct microscopic examination, *n (%)*	33/37 (89)
Culture, *n (%)*	35/37 (94)
Positive RT-PCR, *n (%)*	7/7 (100)
Positive blood culture,* n (%)*	28 (70)
Positive medullar sample	
Direct microscopic examination,* n (%)*	22/37 (59)
Culture,* n (%)*	27/37 (73)
Patients with at least one positive culture,* n (%)*	40 (100)

Results are number *n* (percentage)

### Patients’ characteristics on ICU admission

Table 2 describes the clinical and biological features of the 40 patients.

**Table 2:** Comparison of HIV-infected patients vs patients with other immunosuppressive underlying conditions vs patients without any identified underlying immunodeficiency

	All patients *n* = 40	HIV- infected *n* = 23	Other immunodeficiency *n* = 11	No identified immunodeficiency *n* = 6	*p*-value
Female, *n (%)*	14 (36)	7 (32)	6 (55)	1 (17)	0.272
Age, *years*	56 (43–61)	49 (41–57)	62 (60–70)	57 (46–70)	0.002
SOFA	11 (8–15)	12 (8–16)	12 (9–16)	7 (6–10)	0.260
Geographic origin, French Guyana, *n (%)*	13 (33)	10 (44)	1 (9)	2 (33)	0.134
HAART, *n (%)*	7 (18)	7 (30)	0 (0)	0 (0)	–
Time between					
Symptoms onset and ICU admission, *days*	22 (14–30)	22 (15–29)	15 (10–23)	40 (31–78.)	0.027
Symptoms onset and treatment, *days*	23 (15–40)	22 (16–31)	16 (11–27)	44 (41–79)	0.001
ICU admission and treatment, *days*	1 (0–2)	1 (0–1)	2 (1–5)	3 (0–8)	0.264
Clinical features					
Temperature, *°C*	39 (38– 40)	39 (38–40)	39 (37–39)	38 (38–39)	0.422
Neurological symptoms, *n (%)*	16 (40)	8 (35)	6 (55)	2 (33)	0.602
Gastro intestinal symptoms, *n (%)*	23 (58)	17 (74)	5 (46)	1 (17)	0.026
Respiratory symptoms, *n (%)*	38 (95)	22 (96)	11 (100)	5 (83)	0.351
Biological data					
Lactate, *mmol/L*	3 (2–5)	3 (2–5)	3 (2–4)	2 (2–3)	0.247
Ferritinemia, *UI/L*	35000 (3000–40000)	40000 (22500–43750)	16631 (3012–40000)	1130 (1000–2500)	0.012
Platelets, *G/L*	67 (16–149)	26 (13–102)	76 (27–111)	176 (126–238)	0.028
Creatinine, µmol*/L*	140 (84–374)	111 (84–348)	239 (114–448)	122 (84–191)	0.492
Triglycerides, *mmol/L*	3 (2–3)	3 (3–4)	3 (3–3)	2 (2–2)	0.297
LDH, *UI/L*	662 (400–1054)	1000 (600–1500)	500 (409–840)	252 (223–272)	0.001
CRP, *mg/L*	150 (93–246)	158 (94–234)	200 (112–314)	95 (83–126)	0.205
CD4 count, *mm*^*−3*^	20 (10–27)	20 (10–27)	NA	NA	–
Severe organ involvement					
Acute respiratory failure, *n (%)*	34 (85)	20 (87)	11 (100)	3 (50)	0.024
Shock, *n (%)*	31 (78)	18 (78)	9 (82)	4 (67)	0.756
Coma, *n (%)*	12 (30)	7 (30)	4 (36)	1 (17)	0.802
Hemophagocytosis lymphohistiocytosis, *n (%)*	19 (48)	15 (65)	3 (27)	1 (17)	0.044
Disseminated histoplasmosis, *n (%)*	32 (80)	22 (96)	9 (82)	1 (17)	< 0.001
Advanced life support therapy					
Renal replacement therapy, *n (%)*	22 (55)	14 (61)	6 (55)	2 (33)	0.550
Mechanical ventilation, *n (%)*	31 (78)	19 (83)	8 (73)	4 (67)	0.655
Outcome					
30-day mortality, *n (%)*	21 (53)	14 (61)	3 (27)	4 (67)	0.151
Length of ICU stay (days)	6 (4–11)	6 (4–9)	6 (4–12)	8 (3–22)	0.796

Results are median (25th–75th quartiles) for continuous variables and number *n* (percentage) for categorical variables

*HIV* human immunodeficiency virus; *SOFA* Sequential Organ Failure Assessment; *HAART* highly active antiretroviral therapy; *LDH* lactate dehydrogenase; *CRP* C-reactive protein; *ICU* intensive care unit

Patients were mostly men (*n* = 26, 65%) with a median age of 56 years (44–62), and SOFA was 11 [8–15] at ICU admission. HIV was the most frequent underlying disease (*n* = 23, 58%), among whom 30% (*n* = 7/23) were receiving highly active antiretroviral therapy (HAART). All HIV patients had a CD4 cell count < 100/mm^3^. Another immunodeficiency was identified in 11 patients (28%), steroid treatment in 23% (*n* = 9), azathioprine in 3% (*n* = 1), SOT in 13% (*n* = 5) and hematological malignancy in 8% (*n* = 3). Of note, all SOT patients were kidney recipients and were transplanted for three years in median. No underlying immunodeficiency could be identified in the remaining 6 patients (15%); notably, all but 1 had diabetes.

Duration of symptoms before ICU admission was 23 days [15–30] and 48% of the patients were hospitalized in general ward before ICU transfer for 8 days [3–13]. Within 24 h of ICU admission, respiratory symptoms were present in 95% of patients (*n* = 38), and most patients had pulmonary abnormalities on CT scan when performed (*n* = 34, Fig. 1, panel C), with a miliary pattern (*n* = 31, 77%), macro-nodules (*n* = 8, 20%), and abnormal lymph nodes (*n* = 6, 15%). Neurological manifestations consisted in altered state of consciousness (30%, *n* = 12), encephalopathy (10%, *n* = 4), and focal deficit (5%, *n* = 2). Cerebral CT was performed in 40% of the patients (*n* = 14) and cerebrospinal fluid analysis in 3 patients and were both normal in all of them. Due to thrombopenia, lumbar puncture was not performed in 9 patients. Brain MRI was performed in 2 patients, without pathological findings. Gastrointestinal involvement was described in 58% (*n* = 23) of the patients. Symptoms were aspecific, mostly diarrhea (*N* = 20, 50%) and abdominal pain (*N* = 7, 18%). Four patients had colitis features on CT. No specific cutaneous manifestations were reported.

Table 2 shows the main biological features on ICU admission. Concomitant bacterial infection at ICU admission was diagnosed in 20% (*n* = 8) of the patients (Additional file 1: Table S1).

### Severe organ involvement and advanced life support therapy

Within 24 h following ICU admission, acute respiratory failure was reported in 85% of the patients (*n* = 34), shock in 78% (*n* = 31), and coma in 30% (*n* = 12). In patients with shock during the 24 h following ICU admission, norepinephrine posology was 1.0 µg/kg/min (0.3–1.5). Hemophagocytosis lymphohistiocytosis was diagnosed in 19 patients (48%) (Additional file 1: Table S2). 80% of the patients (*n* = 32) had disseminated histoplasmosis, and 20% had pulmonary histoplasmosis.

Of the patients, 78% (*n* = 31) were mechanically ventilated, with a P/F ratio of 110 mm Hg (75–200) at MV onset. Renal replacement therapy was initiated in 55% of patients (*n* = 22). Among survivors, all were weaned from renal replacement therapy at ICU discharge.

### Differences according to the underlying immunodeficiency

HLH was more frequent in HIV-positive patients than in the two other groups. Patients without identified underlying immunodeficiency were characterized by a lower prevalence of disseminated histoplasmosis (*p* < 0.001) and a longer time from symptom onset to treatment (*p* = 0.001) than that observed in HIV-infected patients and in those with another immunodeficiency.

### Treatment and outcome

Antifungal therapy was given before the diagnosis of histoplasmosis to 15% of patients (n = 6) and after the diagnosis to 85% (*n* = 34). Liposomal amphotericin B (dose 5 mg/kg/day) was used in 80% of the patients (*n* = 32) and itraconazole in 20% (*n* = 8). All survivors received a 14-day treatment course with a switch to itraconazole as maintenance therapy. Time from symptom onset to treatment was 23 days (15–40), and time from ICU admission to treatment was 1 day (0–2). Of note, no patients had antifungal prophylaxis before hospital admission. Once the diagnosis of histoplasmosis was confirmed, all patients received steroids (40 mg/day of prednisone).

**Fig. 2:**
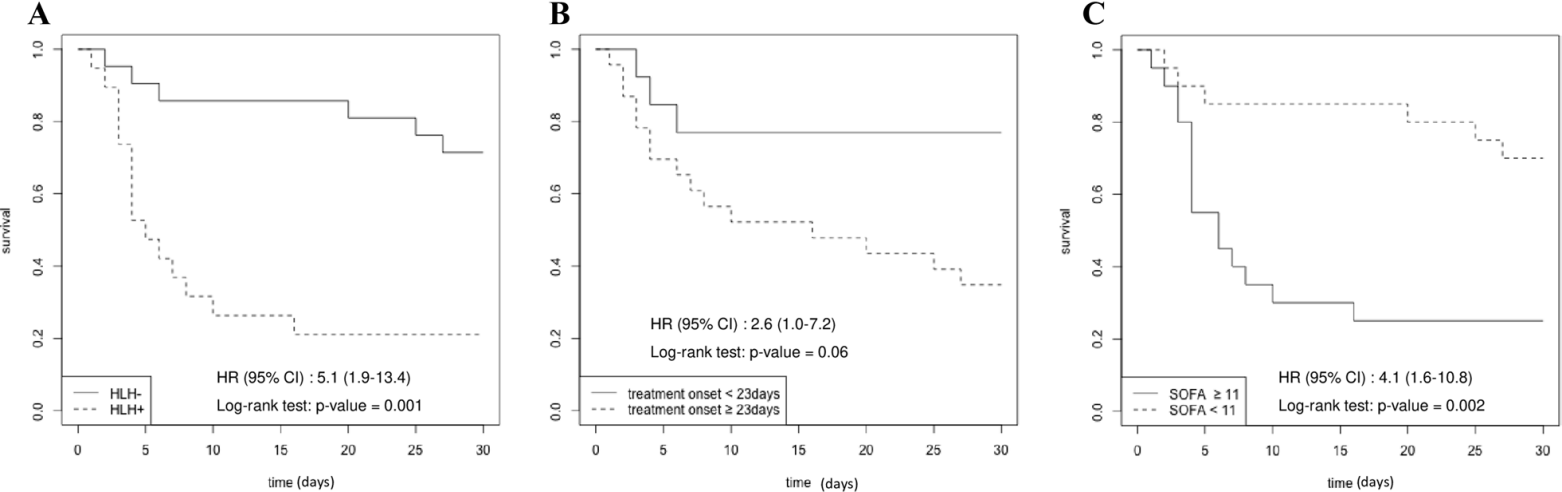
Kaplan–Meier survival estimates according to the presence or not of hemophagocytic lymphohistiocytosis (HLH, **A**), time from symptom onset to treatment (more or less than 23 days, **B**), and severity on ICU admission (Sequential Organ Failure Assessment; [ SOFA] score below vs above or equal 11, **C**)

The 30-day mortality was 53% (*n* = 21). Table 3 shows the factors associated with 30-day mortality identified by univariate analysis. By multivariate analysis, three factors were independently associated with 30-day mortality: SOFA score (OR 1.5, 95% CI 1.1–2.1, *p* = 0.01), time between symptoms onset and treatment, per day (OR 1.1, 95% CI 1.0–1.1, *p* = 0.02), and HLH (OR 6.4, 95% CI 1.1–47.5, *p* = 0.05) (Table 3). Factors associated with 30-day mortality identified by multivariate analysis performed only in immunocompromised patients were time between Hospital admission and treatment, lactate, SOFA score and HLH (Additional file 1: Table S3). Figure 2 shows Kaplan–Meier survival estimates according to the presence or not of HLH, time from symptom onset to treatment (more or less than 23 days), and severity on ICU admission (SOFA score below vs. above or equal 11).

**Table 3:** Factors associated with 30-day mortality by univariate and multivariate analysis

	30-day survivors (*n* = 19)	30-day non-survivor (*n* = 21)	Univariate analysis		Multivariate analysis	
			OR (95%CI)	*p*	OR (95% CI)	*p*
Female, *n (%)*	8 (44)	6 (29)				
Age, *years*	57 (46–61)	55 (41–62)	1 (1.0–1.0)	0.882		
SOFA score	8 (6–11)	14 (10–16)	1.3 (1.1–1.6)	0.001	1.5 (1.1–2.1)	0.01
Geographic origin, French Guyana, *n (%)*	5 (33)	8 (40)	1.3 (0.3–5.8)	0.707		
Time between						
Symptom onset and ICU admission, *days*	15 (10–28.5)	28 (20–34)	1.0 (1.0–1.1)	0.078		
Symptom onset and treatment, *days*	16 (11–32)	29 (21–40)	1.0 (1.0–1.1)	0.078	1.1 (1.0–1.1)	0.02
ICU admission and treatment, *days*	1 (0–2)	2 (0–4)	1.1 (0.9 –1.3)	0.139		
Underlying immunodeficiency						
HIV-infected, *n (%)*	9 (47)	14 (67)	–	0.151		
Other immunodeficiency,* n (%)*	8 (42)	3 (14)	0.26 (0.0–1.2)			
No identified immunodeficiency,* n (%)*	2 (11)	4 (19)	1.2 (0.2–11.7)			
Clinical features						
Temperature, *°C*	39 (38–40)	39 (38–40)	0.9 (0.6–1.5)	0.891		
Neurological symptoms, *n (%)*	5 (26)	11 (52)	3.0 (0.8–12.4)	0.175		
Respiratory symptoms, *n (%)*	19 (100)	19 (90)		0.488		
Gastrointestinal symptoms, *n (%)*	11 (58)	12 (57)	1.0 (0.3–3.5)	1.0		
Biological data						
Lactate, *mmol/L*	2 (2 –3)	3 (2 –5)	1.6 (1.0–2.4)	0.035		
Ferritinemia, *UI/L*	10000 (3000–35000)	40000 (40000–47500)	1.0 (1.0–1.0)	0.012		
Platelets, *G/L*	92 (54–205)	26 (12–96)	1.0 (1.0–1.0)	0.096		
Creatinine, µmol */L*	102 (70–344)	206 (110–476)	1.0 (1.0–1.0)	0.110		
Triglycerides, *mmol/L*	2.5 (2.3–4.0)	3.1 (2.9–3.3)	1.1 (0.5–2.8)	0.569		
LDH, *UI/L*	500 (400–935)	989 (468–1224)	1.0 (1.0–1.0)	0.317		
CRP, *mg/L*	150 (87–222)	146 (95–255)	1.0 (1.0–1.0)	0.910		
Severe organ involvement						
Acute respiratory failure, *n (%)*	16 (84)	18 (86)	1.1 (0.2–7.4)	1.0		
Shock, *n (%)*	10 (53)	21 (100)		< 0.001		
Coma, *n (%)*	3 (16)	9 (43)	3.8 (0.9–21.1)	0.128		
Hemophagocytic lymphohistiocytosis, *n (%)*	4 (21)	15 (71)	8.6 (2.1–42.6)	0.004	6.4 (1.1–47.5)	0.05
Disseminated histoplasmosis, *n (%)*	15 (79)	17 (81)	1.1 (0.2–5.3)	0.878		
Advanced life support therapy						
Renal replacement therapy, *n (%)*	6 (32)	16 (76)	6.5 (1.7–29.2)	0.006		
Mechanical ventilation, *n (%)*	10 (53)	21 (100)		< 0.001		

Results are median (25th–75th quartiles) for continuous variables, number n (percentage) for categorical variables, and odds ratio (OR) and 95% confidence interval (CI)

*HIV* human immunodeficiency virus; *SOFA* Sequential Organ Failure Assessment; *LDH* lactate dehydrogenase; *CRP* C-reactive protein; *ICU* intensive care unit

## Discussion

Histoplasmosis is a protean disease leading to multiple organ failure (MOF). In our study, the prognosis was grim, being fatal in 53% of patients, and was influenced by illness severity and the time between symptom onset and treatment. Interestingly, delay in treatment initiation was greater among patients without underlying immunodeficiency (Table 3). In endemic areas, the prevalence of histoplasmosis remains unknown [21] and common beliefs about this disease (based on old data) are changing. In large historical series [22–24], life-threatening events reported were respiratory, hemodynamic, and neurological failures. However, ICU access was heterogeneous in these cohorts. To the best of our knowledge, no large cohort of critically ill histoplasmosis patients has yet been published.

In our retrospective study performed in ICUs in two endemic areas, critically ill histoplasmosis patients represented 1–3 cases per year for each center. Most of the patients were immunocompromised (85%), but 15% had no identified immunodeficiency. In a recent North American epidemiologic study on predisposing factors for histoplasmosis [2, 25], immunosuppressive drugs were the main risk factors, differing from our study in which HIV was the main underlying condition (58% of the patients). This could be explained by socio-economic differences between North America and our study areas. Histoplasmosis diagnosis is challenging [2], as shown by the long duration of symptoms before treatment in our study, and is still based on the experience of both the physician and the mycologist. Main determinants of this long time could be the poor specificity of histoplasmosis symptoms associated with a misknowledge of the disease. Median time for treatment initiation in the ICU was only 1 day in our study, similar to previous results [25]. This rather short time [14, 23, 26] could result from two factors: (1) the predominance of disseminated histoplasmosis with a large fungal load; and (2) the experience of the intensivists in an endemic area.

Some specific characteristics of histoplasmosis should be highlighted to help physicians in the diagnosis process. In our series, thoracic CT showed a predominantly miliary pattern. The diagnostic yield of BAL analysis (Table 2) was high (positive in 88% of cases), probably influenced by the high frequency of pulmonary involvement. The diagnostic yield of bone marrow aspirate samples was also high (73%) and, interestingly, identical in HIV-infected and non-HIV-infected immunocompromised patients, differing from previous results [27]. Of note, patients without any identified underlying immunodeficiency had a higher rate of pulmonary histoplasmosis (83%) than the other groups. During the ICU course, patients had severe MOF, with a high prevalence of mechanical organ replacement. Respiratory involvement was severe, as shown by the high need for mechanical ventilation and major hypoxemia at MV onset. The high prevalence of catecholamine need (77% of the patients) is consistent with the described frequency of severe hypotension in histoplasmosis (up to 10% of the patients in a large HIV cohort [15]). Shock was previously thought to be secondary to bacterial coinfections in histoplasmosis. In our cohort, 20% of patients had bacterial coinfection at admission. We were surprised to find only two opportunistic infections at ICU admission. This is in contrast with historical series, in which concomitant opportunistic infections were found at histoplasmosis diagnosis in up to 70% of patients [5, 22, 28]. Therefore, we think that shock is a common feature of severe histoplasmosis.

Acute kidney injury (AKI) is frequently associated with histoplasmosis (in up to 59% of patients [15]). The etiology of histoplasmosis-AKI is believed to be multifactorial (4). In our cohort, the severity of AKI was high, as 55% (*n* = 22) of affected patients required RRT.

Overall mortality was consistent with rates reported for severe histoplasmosis in AIDS patients [4, 22]. However, those studies were done before the widespread use of HAART, and ICU access was heterogeneous. In a recent cohort of AIDS patients under HAART, histoplasmosis prognosis remains dire with a mortality rate of 12% [22]. In patients under anti-TNF therapy and SOT recipients [26, 29], prognosis was reported to be better. Interestingly, we found that prognosis seemed similar for HIV patients and other immunosuppressed patients, differing from previous comparative reports [23]. Strikingly, patients without underlying immune disorders had a high mortality rate (67%, *n* = 4). This was probably secondary to the long time between symptom onset and treatment initiation for this sub-population (Table 3 and Fig. 2).

In histoplasmosis, factors associated with mortality have been studied only in AIDS patients [4, 22, 28]. In univariate analysis, we identified that MV, shock, need for Renal Replacement Therapy, time between symptoms onset and treatment, and HLH were associated with death, both for HIV- and non-HIV-immunosuppressed patients (Table 3). These factors, but not HLH are consistent with the data of large AIDS cohorts [4, 22, 28]. The burden of renal failure and shock was not described for non-HIV patients in those studies.

In multivariate analysis, clinical severity reflected by SOFA score, delay of treatment initiation, and HLH were independently associated with death (Fig. 2). The weight of HLH on histoplasmosis prognosis was previously discussed [4, 30], but remained unproved. This could be due to an underestimation of HLH frequency, as medullar analysis is missing in most studies [22, 28, 30]. In the ICU, HLH bears a grim prognosis with a high case-fatality rate largely influenced by the etiology of HLH [31]. Adjunctive therapy for HLH secondary to histoplasmosis has been reported [30, 32], with seemingly good tolerance. None of our patients had adjunctive therapies for HLH, apart steroid according to guidelines [9]. Etoposide use in severely immunocompromised patients with histoplasmosis need to be evaluated due to potential infectious side-effects [33].

No biological markers, including those associated with HLH, were predictive of death (as previously described [4, 12, 22]).

Considering the severity of histoplasmosis in the ICU and the impact of delay in treatment on mortality, this diagnosis should be systematically raised in endemic areas in case of lung miliary lesions, micronodules, or an excavated lesion. In these areas, we suggest adding systematically empirical antifungal treatment in case of pulmonary miliary lesions with shock (as recommended by others [15], especially in immunosuppressed patients). In our serie, 4 patients were diagnosed with histoplasmosis post mortem (all had pulmonary miliary lesions and shock).

Our study has several limitations. First, due to its small sample size, the results of the multivariate analysis should be interpreted with caution. Second, we studied a mixed population of HIV-infected patients, patients with other immunosuppressive underlying conditions (SOT, hemopathy), and patients without any identified immunodeficiency who had their own contributing characteristics. No specific analysis of factors associated with 30-day mortality in each patient subgroup could be performed, due to the subgroups’ sizes. Third, due to the retrospective study design, selection bias could have occurred, and we were not able to estimate the prevalence of severe histoplasmosis in the two endemic areas. Fourth, as it is informed by patients, time from symptoms onset to hospitalization might be unprecise. However, historical series of histoplasmosis cases were also small in size due to the rarity of the disease. Due to the multicentric nature of our study in two large endemic areas, we think that our data may be consistent with the characteristics of histoplasmosis patients treated in the ICU.

## Conclusion

In endemic areas, severe histoplasmosis is not uncommon in the ICU. Critically ill patients with histoplasmosis are mostly immunocompromised, with disseminated forms, leading to multiorgan failure. Patients with no underlying immunodeficiency are also at risk. The overall prognosis seems grim, even with appropriate antifungal therapy. Multiple organ failure, hemophagocytic lymphohistiocytosis, and delay of treatment initiation are the main identified risk factors for death.

## Supplementary Information

**Additional file 1: Table S1**. Concomitant infection at ICU admission. **Table S2**. Clinical and Biological features of patients with Hemophagocytosis lymphohistiocytosis at ICU admission. **Table S3**. Factors associated with 30-day mortality by univariate and multivariate analysis after excluding patients with no identified immunodeficiency.

## Data Availability

All data and materials are available on demand.
