# Supplementary material for: Factors related to mortality in critically ill histoplasmosis: a multicenter retrospective study in Guadeloupe and French Guyana
Source: Ann Intensive Care. 2023 Apr 21;13:30. doi: 10.1186/s13613-023-01128-7 (PMC10121956; doi:10.1186/s13613-023-01128-7)
Supplement: Supplementary file 1 — Additional file 1: Table S1. Concomitant infection at ICU admission. Table S2. Clinical and Biological features of patients with Hemophagocytosis lymphohistiocytosis at ICU admission. Table S3. Factors associated with 30-day mortality by univariate and multivariate analysis after excluding patients with no identified immunodeficiency. [file 13613_2023_1128_MOESM1_ESM.docx]

**Additional Tables**

**Table S1: concomitant infection at ICU admission**

|  |  |
| --- | --- |
| Case 1 | Pneumonia with bacteriemia due to *Pseudomonas aeruginosa*, |
| Case 2 | Pneumonia due to *methicillin sensitive Staphylococcus aureus* |
| Case 3 | Cerebral nocardiosis |
| Case 4 | *Klebsiella pneumoniae* Bacteriemia |
| Case 5 | *Pseudomonas aeruginosa* Bacteriemia |
| Case 6 | *Escherichia coli* Bacteriemia |
| Case 7 | Pneumonia due to *Klebsiella pneumoniae* |
| Case 8 | Tuberculosis of the central nervous system |
|  | |

**Table S2: Clinical and Biological features of patients with Haemophagocytosis lymphohistiocytosis at ICU admission**

|  | N = 19 |
| --- | --- |
| Female, *n (%)* | 5 (26) |
| Age, *years* | 50 (47-62) |
| SOFA | 14 (12-16) |
| Geographic origin, French Guyana, *n (%)* | 6 (30) |
| **Time between** |  |
| Symptoms onset and ICU admission, *days* | 27 (18-30) |
| Symptoms onset and treatment, *days* | 28 (21-32) |
| ICU admission and treatment, *days* | 1 (0-2) |
| **Underlying immunodeficiency** |  |
| HIV-infected, *n (%)* | 15 (79) |
| Other immunodeficiency, *n (%)* | 3 (16) |
| No identified immunodeficiency, *n (%)* | 1 (5) |
| **Clinical features** |  |
| Temperature, *°C* | 39.0 (38.7 – 40.0) |
| Neurological symptoms, *n (%)* | 10 (53) |
| Gastro intestinal symptoms, *n (%)* | 12 (63) |
| Respiratory symptoms, *n (%)* | 18 (95) |
| **Biological data** |  |
| Lactate, *mmol/L* | 3.9 (2.3-5.7) |
| Ferritinemia, *UI/L* | 40000 (37500-47500) |
| Platelets, *G/L* | 20 (11-58) |
| Hemoglobin, g/dL | 8.5 (7.6-9.8) |
| Leukocytes, G/L | 3.8 (2.2-5.9) |
| Creatinine, µmol*/L* | 231 (98-386) |
| Triglycerides, *mmol/L* | 3.3 (3.0-3.7) |
| LDH, *UI/L* | 1025 (832-1875) |
| CRP, *mg/L* | 231 (121-320) |
| HScore | 197 (158-232) |
| **Severe organ involvement** |  |
| Acute respiratory failure, *n (%)* | 16 (84) |
| Shock, *n (%)* | 17 (90) |
| Coma, *n (%)* | 9 (47) |
| Disseminated histoplasmosis, *n (%)* | 18 (95) |
| **Advanced life support therapy** |  |
| Renal replacement therapy, *n (%)* | 15 (79) |
| Mechanical ventilation, *n (%)* | 16 (84) |
| **Outcome** |  |
| 30-day mortality, *n (%)* | 15 (79) |
| Length of ICU stay (days) | 5 (4-9) |

Results are median (25^th^–75^th^ quartiles) for continuous variables and number n (percentage) for categorical variables. HIV, human immunodeficiency virus- SOFA, Sequential Organ Failure Assessment- LDH, lactate dehydrogenase- CRP, C-reactive protein- ICU, intensive care unit.

**Table S3. Factors associated with 30-day mortality by univariate and multivariate analysis after excluding patients with no identified immunodeficiency**

|  | 30-day survivors  (n = 17) | 30-day non-survivor  (n = 17) | Univariate analysis | | Multivariate analysis | |
| --- | --- | --- | --- | --- | --- | --- |
|  |  |  | OR (95%CI) | p | OR (95% CI) | p |
|  |  |  |  |  |  |  |
| Female, *n (%)* | 7 (44) | 6 (35) |  | 0.888 |  |  |
| Age, *years* | 58 (49-61) | 49 (40-60) | 1.0 (0.9-1.0) | 0.241 |  |  |
| SOFA score | 8 (7-12) | 15 (12-17) | 1.4 (1.1-1.8) | 0.001 | 1.5 (1.1–2.2) | 0.023 |
| Geographic origin, French Guyana, *n (%)* | 6 (35) | 5 (29) | 0.8 (0.2-3.4) | 1.000 |  |  |
| **Time between** |  |  |  |  |  |  |
| Symptom onset and ICU admission, *days* | 15 (10-27) | 22 (15-30) | 1.0 (1.0-1.1) | 0.209 |  |  |
| Symptom onset and treatment, *days* | 16 (12-31) | 29 (21-32) | 1.0 (1.0-1.1) | 0.097 |  |  |
| ICU admission and treatment, *days* | 1 (0-2) | 2 (0-2) | 1.0 (0.8-1.2) | 0.463 |  |  |
| Hospital admission and treatment, *days* | 2 (0-5) | 6 (1-14) | 1.1 (1.0-1.2) | 0.082 | 1.2 (1.1-1.6) | 0.028 |
| **Clinical features** |  |  |  |  |  |  |
| Temperature, *°C* | 38.5 (37.5-40.0) | 39.0 (37.7-39.5) | 1.1 (0.7-1.9) | 0.638 |  |  |
| Neurological symptoms, *n (%)* | 5 (29.4) | 9 (53) | 2.6 (0.6-11.7) | 0.296 |  |  |
| Respiratory symptoms, *n (%)* | 17 (100) | 16 (94) |  | 1.000 |  |  |
| Gastrointestinal symptoms, *n (%)* | 11 (65) | 11 (65) | 1.0 (0.2-4.3) | 1.000 |  |  |
| **Biological data** |  |  |  |  |  |  |
| Lactate, *mmol/L* | 3 (1-4) | 4 (3-6) | 1.6 (1.0-2.6) | 0.040 | 1.4 (0.96-3.2) | 0.029 |
| Ferritinemia, *UI/L* | 10000 (3000-35000) | 40000 (40000-47500) | 1.0 (1.0-1.0) | 0.002 |  |  |
| Platelets, *G/L* | 85 (44-210) | 20 (12-54) | 1.0 (1.0-1.0) | 0.021 |  |  |
| Creatinine, µmol */L* | 127 (70-347) | 231 (110-476) | 1.0 (1.0-1.0) | 0.256 |  |  |
| Triglycerids, *mmol/L* | 2 (2-4) | 3 (3-3) | 1.4 (0.5-3.9) | 0.360 |  |  |
| LDH, *UI/L* | 600 (450-968) | 1025 (710-1625) | 1.0 (1.0-1.0) | 0.081 |  |  |
| **Severe organ involvement** |  |  |  |  |  |  |
| Acute respiratory failure, *n (%)* | 15 (88) | 16 (94) | 2.0 (0.1-66.6) | 1.0 |  |  |
| Shock, *n (%)* | 10 (59) | 17 (100) |  | 0.007 |  |  |
| Coma, *n (%)* | 3 (18) | 8 (47) | 3.9 (0.8-22.9) | 0.143 |  |  |
| Hemophagocytic lymphohistiocytosis, *n (%)* | 4 (24) | 14 (82) | 13.2 (2.7-88.2) | 0.002 | 6.3 (0.9–66) | 0.084 |
| Disseminated histoplasmosis, *n (%)* | 14 (82) | 17 (100) |  | 0.227 |  |  |
| **Advanced life support therapy** |  |  |  |  |  |  |
| Renal replacement therapy, *n (%)* | 6 (35) | 14 (82) | 7.7 (1.7-47.2) | 0.015 |  |  |
| Mechanical Ventilation, *n (%)* | 10 (59) | 17 (100) |  | 0.007 |  |  |
|  | | | | | | |

Results are median (25^th^–75^th^ quartiles) for continuous variables, number n (percentage) for categorical variables, and odds ratio (OR) and 95% confidence interval (CI).

HIV, human immunodeficiency virus; SOFA, Sequential Organ Failure Assessment; LDH, lactate dehydrogenase; CRP, C-reactive protein; ICU, intensive care unit
